# Oncolytic virus and immunogenic cell death in cancer therapy

**DOI:** 10.1016/j.tvr.2025.200333

**Published:** 2025-11-26

**Authors:** GuoXiu Cao, Chan Ding, Jun Dai, Xusheng Qiu

**Affiliations:** a Guizhou. Aerospace Hospital, Zunyi, 563000, China; b Shanghai Veterinary Research Institute, Chinese Academy of Agricultural Sciences, Shanghai, 200241, China; c Experimental Animal Center, Zunyi Medical University, Zunyi, 563000, China

**Keywords:** Oncolytic viruses, Immunogenic cell death, Cancer, Therapy

## Abstract

As a promising cancer treatment strategy, oncolytic viruses (OVs) selectively replicate and kill tumor cells while sparing normal cells. They improve the tumor immunosuppressive microenvironment through multiple mechanisms, including direct infection, replication, and lysis of tumor cells—leading to the release of tumor-associated antigens (TAAs), chemokines, and cytokines, which in turn induce immunogenic cell death (ICD) and trigger sustained antitumor immune responses. Currently, while OVs have demonstrated therapeutic efficacy in multiple preclinical and clinical studies, their monotherapy fails to benefit a broad spectrum of cancer patients. Therefore, there remains a need to fully understand the biological mechanisms of OVs and optimize immunotherapeutic strategies to benefit more cancer patients and enhance therapeutic efficacy. In this review, we discuss how the immune responses induced by OVs maintain a balance between antiviral and antitumor immunity, as well as their unique characteristics in inducing ICD. In addition, we describe how to enhance the efficacy of cancer immunotherapy by combining OVs therapy with ICD inducers, aiming to provide valuable insights to guide the development of clinical OVs -based therapies.

## Highlights

• Oncolytic viruses selectively infect tumor cells while sparing normal cells.
• Oncolytic viruses evolve multiple mechanisms to induce immunogenic cell death.
• Engineering oncolytic viruses as potential methods for enhancing antitumor efficacy.
• Oncolytic viruses combined with immunogenic cell death inducers are potential antitumor therapy.

## Graphical abstract

a | OVs selectively infect and replicate in tumor cells while sparing normal cells, modulate the balance between antiviral and antitumor responses, and release tumor-associated antigens to recruit innate effector cells. **b |** In lymph nodes, oncolytic viruses and immunogenic cell death inducers promote the presentation of tumor antigens to naive and memory T cells. **c |** After infiltrating tumor sites, activated T cells recognize tumor antigens, induce immunogenic cell death, and thereby maximize tumor cell elimination.

**Figure:**
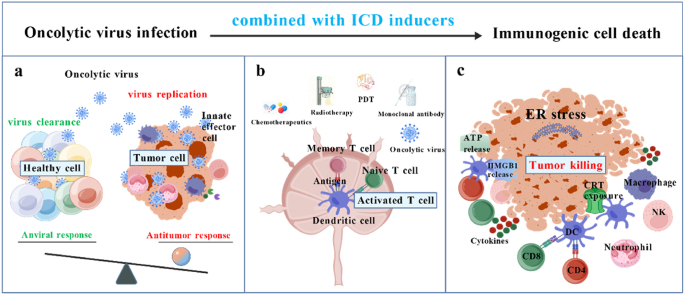

## 1 Introduction

Until the end of the 19th century, surgery was the main cancer treatment. With the rapid development of pathology, cytology, and physiology, new therapies—including chemotherapy, radiotherapy (RT), and immunotherapies—have been introduced, greatly advancing cancer treatment [1]. Over the following century, understanding of viruses has grown [[2], [3], [4]], and their ability to kill cancer cells was confirmed; however, clinical trials only proved viral therapeutic benefits for cancer patients in the past two decades [5,6].

OVs are natural or genetically engineered viruses, classified by nucleic acid type into DNA viruses (e.g., herpes simplex virus [HSV], adenovirus [Ads]) and RNA viruses (e.g., measles virus [MeV], reovirus [RV]) [7]. Functionally, they include wild-type/laboratory-attenuated viruses (naturally tumor-selective, e.g., RV) and genetically modified variants (optimized for safety/efficacy, e.g., ICP34.5-deleted HSV-1) [1,4,8]. As an emerging cancer therapy, OVs uniquely target, infect, replicate in, and kill cancer cells while sparing healthy cells [9] —a key difference from conventional immunotherapies (e.g., immune checkpoint inhibitors [ICIs]), which mainly modulate pre-existing immune responses rather than directly shaping tumor immunogenicity [10]. Notably, OVs act as “in situ vaccines”: by lysing tumor cells and releasing TAAs and damage-associated molecular patterns (DAMPs), they convert immunologically “cold” (poorly immune-infiltrated) tumors into “hot” ones, laying the groundwork for exploring synergies between OVs and other immunogenic cell death (ICD) inducers [11]. Beyond direct lysis, the replicative nature of OVs further promotes local TAA and DAMP release to mediate antitumor immunity [7,12]. Released TAAs serve as immune recognition targets, while DAMPs act as “danger signals” to activate the immune system; together, they trigger activation of antigen-presenting cells (APCs) and naive T cells, drive immune cell infiltration into tumors, and induce persistent antitumor immunity [13,14]. This death process—driven by the release of danger signals (e.g., DAMPs) from dying cells—is defined as ICD [15]. The ICD concept has expanded understanding of tumor immunotherapy, enhances host immunogenicity, and induces tumor-specific responses [16]. Multiple cancer therapies, such as chemotherapy drugs (e.g., anthracyclines, oxaliplatin) [17], RT [18], and photodynamic therapy (PDT) [19], can trigger ICD. Notably, while these therapies activate ICD-related immunostimulatory pathways, their anticancer efficacy—like that of OVs—depends not only on direct effects on cancer cells but also on modulation of host immune function [[20], [21], [22]].

Growing preclinical and clinical evidence supports OV therapy as promising, either as monotherapy or in combination with other treatments [[23], [24], [25], [26]]. In this review, we summarize key mechanisms by which OVs exert antitumor effects via immune microenvironment modulation and ICD pathway regulation. We also describe recent clinical advances in OV combinations with ICD inducers, focusing on how engineered OVs synergize with ICD inducers to enhance antitumor efficacy and benefit more patients.

## 2 Oncolytic virus-mediated immune activation

**Fig. 1:**
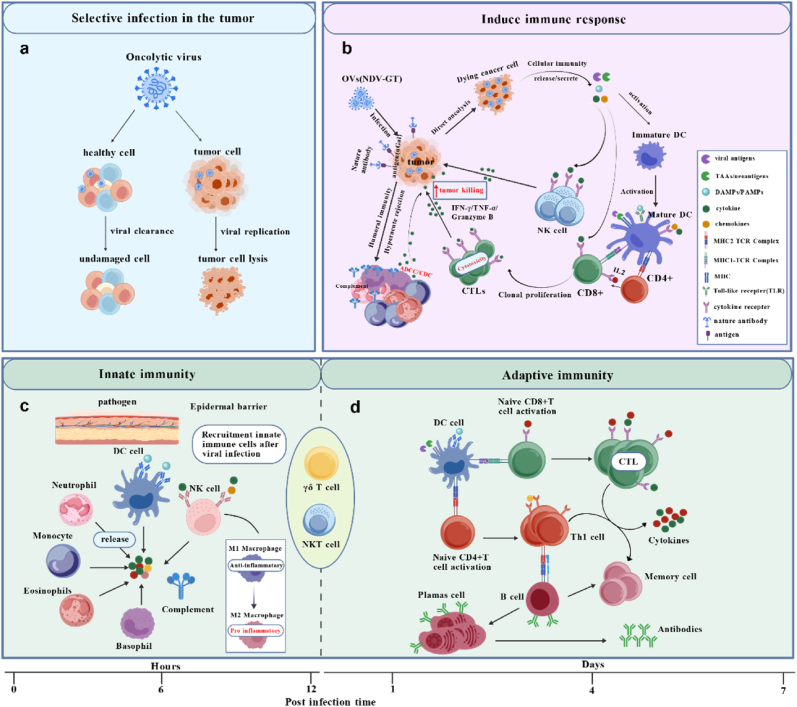
Mechanism of OV-mediated antitumor immune response. The antitumor activity of OVs relies on multiple mechanisms that target the cancer immune cycle [55]. **a |** OVs selectively replicate in, infect, and kill cancer cells (without harming normal cells) [34]. **b |**Post-infecting tumor cells, oncolytic viruses (e.g., NDV-GT) exert dual antitumor effects [274]: Direct lysis of tumor cells releases TAAs, PAMPs/DAMPs [13], and cytokines. These promote maturation of APCs (e.g., DCs) to activate T cells [57,59,75], triggering tumor-specific immunity—inducing cytotoxic CD8^+^ T cell clonal proliferation and pro-inflammatory cytokine release. Activated Natureal kill (NK) cells also secrete pro-inflammatory factors to enhance tumor killing [27]. Certain engineered OVs express antigens that bind antibodies, inducing hyperacute rejection to activate humoral immunity. This mediates ADCC and CDC, amplifying pro-inflammatory cytokine release and promoting tumor cell apoptosis [61]. **c |** OVs cross the skin barrier into the tumor area, infect tumor cells, and release TAAs. Innate immune cells rapidly respond they detect viral membrane molecules to initiate non-specific immunity [275]; phagocytes (neutrophils, eosinophils, macrophages, NK cells, etc.) engulf infected tumor cells, release antigens extracellularly, activate complement, and secrete cytokines to induce inflammation. **d |** Adaptive immunity (slower-acting) is triggered by DCs presenting antigens to T/B cells [71]: naive T cells activate and differentiate into cytotoxic CD8^+^ T cells and Th1 cells (secreting inflammatory cytokines to enhance tumor killing) [276]; B cells are induced to produce antibodies. Memory cells form simultaneously, enabling sustained immune responses for antitumor effects [63]. Figure created with Bio GDP.

OVs kill tumor cells through a dual mechanism of action: selective direct killing of tumor cells, and indirect induction of systemic antitumor immune responses to eliminate residual tumor cells [27]. In this process, OVs activate innate and adaptive immune responses against viruses and tumors through infection [28]. Although the mechanisms of action are incompletely understood, the killing effects of these mechanisms vary depending on the nature and type of cancer cells, the characteristics of viral vectors, and the interactions between the tumor microenvironment (TME) and the host immune system [29]. However, multiple studies have shown that the key to the antitumor effect of OVs lies in the immune response state in the TME and the intensity of immune activation induced by OVs (Fig. 1) [[30], [31], [32], [33]].

### 2.1 Selective replication in tumor cells

Viruses can infect various cells, but OVs selectively target tumor cells: upon entry, normal cells activate antiviral mechanisms to control and clear viral replication, avoiding damage [27,34]. While some viruses (e.g., vaccinia virus [VACV], MeV) naturally target tumors [[35], [36], [37]], others require engineering—such as HSV-1 and engineered Ads, which achieve selective replication in tumor cells via genetic modification of virulence genes [[38], [39], [40]]. Tumor intrinsic properties drive OV selectivity. First, OVs utilizing retargeting strategies (e.g., engineered MeV, Ads) enter cells via tumor-specific receptors (e.g., CD46, ICAM-1, DSG2)—which are absent or low in normal cells [41]; modifying viral proteins can further enhance this targeting [7]. Second, tumor cells accelerated metabolism enables viral hijacking of host machinery for rapid replication [34]. Third, OVs exploit tumor-specific abnormal pathways (e.g., activated Ras, p53 deficiency, impaired IFN-I signaling) [42,43] —for example, Talimogene laherparepvec (T-VEC, FDA-approved oncolytic HSV-1) evades normal cell antiviral responses via γ34.5 gene deletion [4]. Unlike normal cells, which possess an intact type I interferon (IFN-I) signaling pathway to suppress viral replication, tumor cells have defects in their IFN-I signaling [44,45]. Beyond STAT1 mutations [45], these mechanisms include JAK1/2 loss-of-function mutations (common in melanoma) disrupting IFN-I-mediated antiviral signaling [46], and downregulated IRF3/7 (key IFN-I transcription factors) in tumors like non-small cell lung and colorectal cancer [47]. This selective permissiveness allows preferential OV replication in tumors while sparing normal tissues (Fig. 1a); notably, many cancer cells lack intrinsic antiviral mechanisms, further facilitating OV replication by blocking viral clearance [48].

### 2.2 Induction of antitumor activity

Beyond direct oncolysis, OVs further eliminate tumors by inducing systemic antitumor immunity [49]. Through genetic engineering or natural tropism, OVs target tumor surface receptors, replicate rapidly, and induce membrane rupture (oncolysis) [50]. Progeny viruses infect adjacent tumor cells, creating cascading cytotoxicity [27]. Moreover, distinct OVs engage specific cell death pathways: HSV (endoplasmic reticulum stress [51]), Ads (mitochondrial apoptosis via caspase-3/9 [52]), and Newcastle disease virus (NDV, regulated by STAT3 [53])—each enhancing tumor-killing. Post-infection/lysis, TAA release into the TME initiates cellular immunity [7]. Concurrently, DAMPs and cytokines promote antigen presentation via MHC molecules, eliciting potent cytotoxic T lymphocyte (CTL) responses [13,14], and recruit lymphocytes to the tumor site for coordinated killing of infected tumor cells while simultaneously eliminating tumor cells in non-directly infected metastatic lesions, thereby inducing the “abscopal effect” (Fig. 1b) [27,54]. However, while OVs eliminate cancer cells by activating the immune system, they may also induce unavoidable immune tolerance that compromises antitumor efficacy [[55], [56], [57]]. Therefore, the key to achieving optimal antitumor effects lies in activating specific immune responses while overcoming immune tolerance. Studies have shown that although tumor cells can express immunosuppressive surface receptors and secrete anti-inflammatory cytokines to inactivate lymphocytes, OVs can modulate the TME status by altering cytokine secretion profiles and immune cell composition [13,14,27]. These OV-mediated changes trigger TAA release and epitope spreading, thereby enhancing immune surveillance against cancer [[58], [59], [60]]. Furthermore, most OVs currently employed in clinical trials for cancer therapy are recombinant variants [30]. These engineered OVs have significantly overcome the limitations of wild-type viruses by partially mitigating immune tolerance and enhancing antitumor potency (Table 1). In addition to activating cellular immunity to enhance tumor-killing effects, certain recombinant OVs can also initiate humoral immunity to amplify the antitumor response. A representative example is recombinant NDV-GT, which retains NDV's tumor-selective replication and oncolysis while encoding the porcine αGal epitope. Antibodies targeting viral surface antigens can simultaneously trigger B-cell-mediated antibody-dependent cell-mediated cytotoxicity (ADCC) and complement-dependent cytotoxicity (CDC), thereby inducing rapid and robust humoral immune responses that further potentiate systemic antitumor immunity [[61], [62], [63]].

**Table 1:** OVs with genetic modification to enhance antitumor ability.

Virus backbone	Recombinant Strains	Modification	Result
NDV	NDV-MIP3α	Insertion of *MIP-3α cDNA* between the P and M gene	**Enhanced Ability**: Retains NDV-WT's lysis/ICD; attracts/promotes DCs; strengthens systemic immunity, suppresses tumors, reverses microenvironment.
			**Shortcomings**: No long-term data; untested in other tumors/human samples [113].
	NDVhuGM-CSF (MEDI5395)、NDVmuGM-CSF	Encoding *human/murine GM-CSF transgene*	**Enhanced Ability**: Broad oncolysis, modulates immune genes; works in 3 models (boosted by immunotherapies), reprograms TME.
			**Shortcomings**: Poor murine tumor replication; no long-term data; untested in humans [183].
	NDV-GT	Addition the *porcine α1,*3 GT *gene*	**Enhanced Ability**: IV safe, eradicates tumors, 90 % disease control, low immunogenicity.
			**Shortcomings**: No long-term data; untested in more tumors/larger cohorts [61].
Ads	ONYX-015	Attenuation via deletion of the *14.7k gene in E1B-55K gene, E3B gene and rid gene*	**Enhanced Ability**: Safe (no serious AEs, high MTD), some efficacy, immune infiltration.
			**Shortcomings**: Limited efficacy (short TTP); narrow use (gliomas only) [202].
	AxdAdB3	1 *E1A and E1B double restriction* (inherited from parent AxdAdB3); 2. *RGD-fiber modification* (enables integrin-dependent infection)	**Enhanced Ability**: Expands oncolysis to CAR^−^ cells, safe for normal cells, inhibits xenograft tumors.
			**Shortcomings**: Limited to biliary cancers; no long-term data/clinical verification [203].
	TelomeKiller	1 Driven by *hTERT promoter* (tumor-specific replication); 2. Encodes *KillerRed* (generates ROS under green light)	**Enhanced Ability**: Targeted PDT, durable efficacy, eliminates lymph node mets, lowers photosensitivity risk.
			**Shortcomings**: Light-dependent; preclinical only; untested in more tumors [204].
	Oncorine (H101)	*E1B-55kD gene* deletion (attenuated, tumor-selective replication)	**Enhanced Ability**: World's first commercial OV, specific replication and dependent cytotoxicity. **Shortcomings**: Limited to NPC; needs chemo; no other tumor efficacy data [195].
	Ad-Surp-mK5、 Ad-Surp-MnSOD	1 *E1B* deleted; 2. Driven by *Survivin promoter (tumor-specific);* 3. Carry *mK5 or MnSOD*	**Enhanced Ability**: Combined use boosts GC cytotoxicity, selective, induces apoptosis, safe. **Shortcomings**: Limited to GC; preclinical only; needs combination for superiority [205].
	rAd5-SARS2-S1	Expresses *SARS-CoV-2 S1 subunit* (non-replicating)	**Enhanced Immunity**: Induces systemic/mucosal immunity (IN better), protects hamsters, neutralizes variants. **Shortcomings**: Preclinical only; Ad5 pre-immunity risk; no long-term data [206].
	oAd5/35-HF	1 Replaces *Ad5 HVRs 1/5 with Ad35's*; 2. Alters *Ad5 fiber with Ad35* components (chimeric capsid)	**Enhanced Ability**: Evades NABs, boosts infection, maintains titters; broad antitumor activity. **Shortcomings**: Preclinical; no long-term data; untested in rare cancers [207].
HSV	G47Δ	3rd-gen triple-mutated oncolytic HSV-1: *γ34.5/α47* deletions, *E. coli lacZ* insertion; optimized for glioblastoma	**Enhanced Ability**: 84.2 % 1-yr survival; safe, immune-activating; Japan-approved, 6-dose-tolerant. **Shortcomings**: Single-arm, small cohort; MRI confusion; invasive; HSV pre-immunity untested [254].
	T-VEC	1 *γ134.5 gene* deleted (limits replication to tumor cells); 2 *Human GM-CSF gene* inserted (enhances antitumor immunity)	**Enhanced Ability**: 1st FDA-approved OV for melanoma; works alone/with ICIs, activates immunity.
			**Shortcomings**: Melanoma-focused; needs intralesional injection; HSV pre-immunity risk [181].
	NG34ScFvPD-1	Inserts gene encoding *anti-PD-1 ScFv*	**Enhanced Ability**: Dual function, immune activation; synergizes with PI3K inhibitor, no oncolysis loss.
			**Shortcomings**: Preclinical; needs PI3K inhibitor; HSV-1 pre-immunity risk; ovarian-only [83].
	OVH-IFNβ、 OVH-IFNβ-iPKR	1 OVH-IFNβ: HSV-1 with *IFNβ gene* (via CRISPR-V) 2 OVH-IFNβ-iPKR: O*VH-IFNβ* expressing IFNβ, inhibiting *PKR*	**Enhanced Ability**: Balances immunity; strong antitumor effects; rapid engineering via CRISPR-V.
			**Shortcomings**: Preclinical; OVH-IFNβ self-inhibits; HSV-1 pre-immunity risk [211].
	HSV-1dko-B7H3nb/CD3、 HSV-1dko-B7H3nb/mCD3、	1 HSV-1dko-B7H3nb/CD3: carries *B7H3nb/CD3 BsAb gene*; edited via *CRISPR/Cas9+cre-loxp* 2 SV-1dko-B7H3nb/mCD3: carries *murine B7H3nb/mCD3 BsAb*; same editing	**Enhanced Ability**: Dual mechanisms; improves TME; better efficacy vs control; efficient editing, targeted.
			**Shortcomings**: Preclinical; murine-specific strain; HSV-1 pre-immunity risk; no combo data [212].
VACV	TG6050	Encodes *single-chain hIL-12 and full-length anti-CTLA-*4 mAb (two immune effectors)	**Enhanced Ability**: Triple mechanisms; works on cold/hot tumors; remodels TME; safe in monkeys.
			**Shortcomings**: Clinical ongoing; species gap; vaccinia pre-immunity risk [182].
MeV	rMV-SLAMblind	Genetically modified to be *SLAM-blind*; infects via *nectin-4/poliovirus receptor-related 4*	**Enhanced Ability**: Targets nectin-4+ CRC; bypasses EGFR resistance; works in xenografts, low burden.
			**Shortcomings**: Preclinical; nectin-4-dependent; measles immunity risk; no immune data [213].

### 2.3 Activation of innate immunity

Innate immunity is the body's first response to invaders entry. In addition to conventional αβ T cells, natural killer (NK) cells, neutrophils, monocytes, as well as γδ T cells and NKT cells (which bridge innate and adaptive immunity), are also present in the TME in various cancer settings. After infection of tumor cells, OVs selectively replicate and release viral pathogen-associated molecular patterns (PAMPs), DAMPs, and cytokines (e.g., IFN-γ, TNF-α, and IL-1α) to induce ICD [62,[64], [65], [66]]. The PAMPs of OVs (e.g., viral nucleic acids or proteins) are recognized by host cell pattern recognition receptors (PRRs), triggering downstream signaling pathways. PRR activation serves as the initiation point of innate immune responses, providing critical signals for subsequent immune cell recruitment and antigen presentation [67]. The recruited innate immune cells subsequently exert direct antitumor effects. For instance, NK cells eliminate MHC-I-deficient tumor cells through the release of proinflammatory cytokines (e.g., IFN-γ) [68], while simultaneously promoting the polarization of anti-inflammatory M2 macrophages into proinflammatory M1 phenotypes [69]. Other immune cells, such as neutrophils, capture and destroy circulating tumor cells via neutrophil extracellular trap formation (NETosis) [70]. Notably, DAMPs, acting as upstream drivers of the adaptive response, also stimulate the release of cytokines from innate immune cells [15,71]. For instance, high mobility group box 1 (HMGB1) binding to toll-like receptors (TLR4) promotes the release of IL-1β and TNF-α from macrophages. These signals induce dying tumor cells to release "eat me" signals, thereby attracting macrophages to recognize and phagocytose dying tumor cells, further promoting the innate response (Fig. 1c) [14,72]. Through PRR recognition, IFN-I release, and recruitment of innate immune cells, this process establishes a bridge connecting local oncolysis to systemic antitumor immunity, thereby laying the foundation for subsequent T cell responses.

### 2.4 Induction of adaptive immunity

Mature dendritic cells (DCs) act as pivotal hubs bridging innate and adaptive immunity. During OV-induced tumor cell lysis, the release of DAMPs (e.g., HMGB1, adenosine triphosphate [ATP]) and TAAs effectively promotes DC maturation and antigen presentation [34,73,74]. Mature DCs present antigens to T cells via MHC molecules, initiating a cellular immune response [74,75]. Activated CD8^+^ CTLs recognize and eliminate virus-infected tumor cells [76], while CD4^+^ helper T cells enhance CTL function and stimulate B-cell antibody production by secreting cytokines (e.g., IFN-γ, IL-2); these cytokines ultimately mediate tumor cell killing through mechanisms including complement activation (Fig. 1d).

Notably, OV-induced adaptive immune responses exhibit considerable breadth and depth. For instance, Jing et al. demonstrated that Semliki Forest Virus 4 (SFV4)-infected tumor cells induced DC activation and maturation, with the production of pro-inflammatory cytokines and chemokines, closely correlating with the activation of antigen-specific CD8^+^ T cells [14]. Recent single-cell RNA sequencing analysis further revealed that both CD4^+^ and CD8^+^ T cell pathways were significantly upregulated in responders treated with genetically engineered HIV-derived OVs, evidenced by the activation of T cell receptor signaling, increased IFN-γ production, and elevated expression of effector molecules such as CCL5 [77]. Although direct oncolysis is transient [78], activated T cells establish immunological memory, enabling long-term immune surveillance and clearance of residual tumor cells [79,80]. This sustained antitumor effect has been preliminarily validated across various cancer types, including glioblastoma [81], relapsed/refractory metastatic cancer [61], breast and colorectal cancer [82], and ovarian cancer [83].

A representative example is DNX-2401 (RGD-modified Ads), which in a Phase I trial for recurrent glioblastoma (GBM) showed 20 % 3-year survival vs. <5 % historically) and three cases of durable tumor regression (>95 % reduction) [81]. Its efficacy is hypothesized to involve dual mechanisms—intratumoral replication for direct oncolysis and subsequent CD8^+^ T cell infiltration—though the small sample size limits definitive conclusions on CD8^+^ T cell infiltration's relative contribution versus other factors. Importantly, the ability of T cells to recognize diverse antigenic epitopes effectively counteracts immune escape caused by tumor mutations, mechanistically addressing challenges such as tumor heterogeneity and therapy resistance [84,85]. Future research should further focus on enhancing cross-presentation efficiency through viral engineering and optimizing the quality of T cell responses by combining immunomodulatory strategies [74,80], thereby comprehensively improving the clinical efficacy of oncolytic virotherapy.

## 3 Modulating the balance between antiviral and antitumor immunity is critical for OVs to exert oncolytic potency

**Fig. 2:**
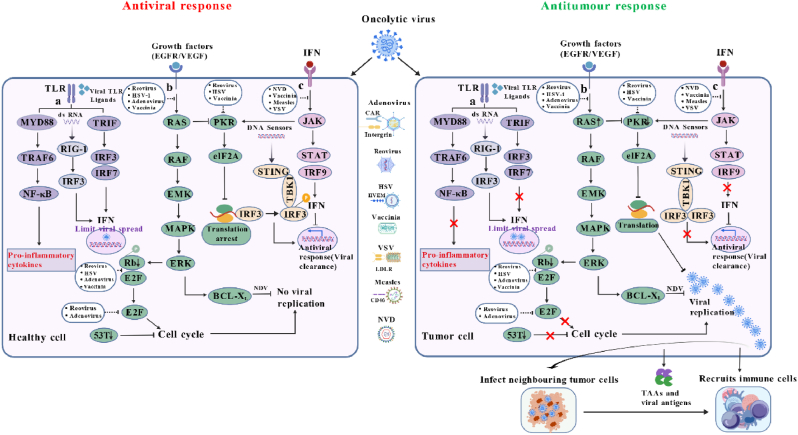
OVs exploit cancer immune evasion and maintain antiviral-antitumor balance. After entering the human body, OVs face distinct responses in healthy cells (left) vs. tumor cells (right): healthy cells use intact intracellular antiviral mechanisms to clear viruses and inhibit replication, while tumor cells cannot, enabling viral lysis and antitumor immunity. **a |** In healthy cells, viral detection activates the TLR pathway: MYD88 triggers NF-κB to induce pro-inflammatory cytokines [15]; RIG-1 and TRIF regulate IRF to drive programmed transcription, limiting viral spread [88,277,278]. In tumor cells, oncogene activity/tumor suppressor loss disrupts TLR-mediated pro-inflammatory cytokine release and TRIF-dependent type I IFN production, failing to restrict viruses [96,102]. **b |** The epidermal growth factor pathway promotes viral replication via RAS-mediated ERK activation [106,108]; PKR induces translation arrest via eIF2A to initiate antiviral responses [107]. In tumor cells, inhibited E2F (downstream of epidermal growth factor pathway) and p53 disrupt cell cycle regulation. **c |** Innate immunity-induced local IFN clears viruses via IFNR [91]: STAT and IRF9 drive type I IFN production [279]; JAK mediates STING-dependent IFN-I release and regulates PKR to trigger translation arrest/antiviral responses [115,118]. Tumor cells downregulate IRF3/IRF9 (IFN-I pathway), impairing antiviral function and virus clearance [116]. Thus, tumor cells are more susceptible to OVs-mediated lysis. Replicated viruses infect adjacent tumors and release viral PAMPs, cellular DAMPs, and pro-inflammatory cytokines, recruiting immune cells to enhance tumor killing [59,87]. Figure created with Bio GDP.

In general, viruses are cleared through two pathways: early antiviral clearance mediated by NK cells and viral elimination via antigen presentation to lymphocytes by mature APCs. These processes collectively limit viral infection and replication, thereby reducing antitumor efficacy [86]. Upon infection of normal cells, TLRs—which detect viral PAMPs and cell-derived DAMPs—are present on the cell surface and in intracellular compartments, activating multiple signaling pathways to clear pathogenic viruses [15]. Concurrently, released cytokines such as IFN-I and TNF, together with STING (a mediator of cytokine induction), activate relevant pathways to coordinate antiviral responses in infected cells, ultimately blocking protein synthesis [27,[87], [88], [89]]. However, in cancer cells, viral clearance pathways are frequently impaired due to mutations or downregulation of key components: such as defective IFN-I signaling (loss of IFNAR/STAT1 function), muted TLR responses, or STING inactivation [29,90,91]. These deficiencies disable the antiviral machinery, allowing OVs to evade clearance, replicate persistently, and exert oncolytic effects while initiating specific antitumor immunity (Fig. 2).

### 3.1 TLRs

TLRs, the first characterized PRRs, recognize diverse pathogens [92]. OVs activate innate immunity via TLRs, with dual antitumor roles: promoting antiviral responses to control viral replication and activating antitumor immunity to eliminate cancer cells—this balance is key for clinical efficacy [34,93]. For example, TLR3/7-mediated IFN-β restricts viral spread [94], while TLR9-driven DC maturation boosts tumor-specific T cells [95]. TLRs also mediate immunity post-OV infection [64,96]. Upon PAMP/DAMP activation, their Toll/IL-1 receptor (TIR) domain recruits signaling adaptors (e.g., MyD88, TRIF, TIRAP) [90,97,98]. Ligand-bound TLRs trigger downstream cascades, establishing a dynamic antiviral-antitumor balance [27]. For instance, TLR3/7 sense viral dsRNA/ssRNA, activate IRF3/7/NF-κB via MyD88/TRIF, and induce IFN-α/β and TNF-α/IL-6 [99,100]; IFN-β then autocrinally suppresses viral replication in normal cells to prevent systemic spread. Excessive TLR signaling disrupts immune homeostasis [101,102], while insufficient signaling fails to activate antitumor immunity or control viral replication. Engineered OVs fine-tune this balance: those expressing TLR-antagonizing peptides (e.g., TLR4 extracellular domain-targeting peptide) reduce excessive activation [103]; those with TLR-regulatory miRNAs (e.g., MyD88-targeting miR-146a) downregulate adaptors [104]. Blocking TLR-ligand binding or intracellular signaling is an effective salvage strategy (Fig. 2a) [96,105].

### 3.2 Growth factors

The epidermal growth factor receptor (EGFR) is a transmembrane receptor tyrosine kinase (RTK) that maintains tissue/cellular signaling and serves as a regulatory target for oncogenic signaling [106]. In healthy cells, OVs entering via growth factor receptors activate RAS/PKR pathways to inhibit protein translation; in tumor cells, PKR activation is blocked, making cancer cells more susceptible to OVs (Fig. 2b) [107,108]. Additionally, vascular endothelial growth factor (VEGF) suppresses IFN-I via ERK1/2 signaling, inactivating antiviral mechanisms and promoting OV replication [109,110]; such as NDV targets ERK downstream of cancer-overexpressed B cell lymphoma-XL (BCL-XL) to prevent apoptosis, ensuring viral replication, propagation, and syncytia formation for dissemination [111]. Beyond EGFR/VEGF-mediated effects, growth factors induce an IFN-γ feedback loop: IFN-γ activates antiviral genes (e.g., PKR, OAS) via STAT1 to limit normal cell infection [112], but excessive IFN-γ suppresses intratumoral OV replication [91]. Clinically, engineered NDV-MIP3α (e.g., insertion of MIP-3α cDNA between the P and M genes) fine-tunes this balance [113].

### 3.3 Type I IFN

IFNs, including IFN-α/β and other less-studied subtypes (e.g., IFN-ε/κ/ω), are produced via PRR activation and mediate antiviral/antitumor responses [91]. After OV entry, local IFN-I release activates PKR—an intracellular kinase recognizing dsRNA and other viral components [114,115]. Activated PKR terminates protein synthesis and promotes viral clearance; however, cancer cells have reduced IFN-I expression and blocked downstream signaling, impairing viral clearance [116,117]. Furthermore, IFN-α/β first binds to IFN-I receptor before recruiting Janus kinase 1 (JAK1) and tyrosine kinase 2 (TYK2), which then activate signal transducer and activator of transcription (STAT) proteins, forming STAT1-STAT2 heterodimers that translocate to the nucleus, associate with interferon regulatory factor 9 (IRF9) to form the interferon-stimulated gene factor 3 (ISGF3) complex, and regulate antiviral responses [44,118]. In summary, OVs selectively evade IFN-I responses in cancer cells—explaining why they are cleared in healthy cells but persist in tumors (Fig. 2c) [44,117]. Additionally, IFNs upregulate antigen processing/presentation: by suppressing host cell transcription while enhancing MHC-mediated presentation of viral peptides, they promote adaptive antitumor immunity [119], thus linking IFN's antiviral function to antitumor efficacy.

## 4 Oncolytic virus-induced immunogenic cell death

**Fig. 3:**
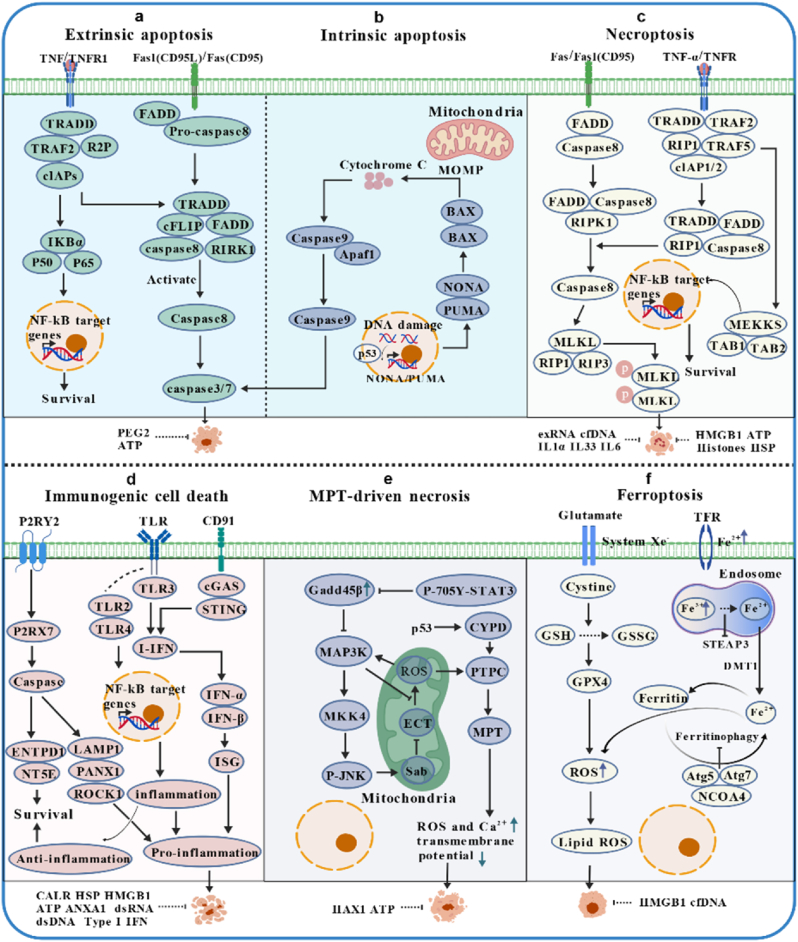
Cell death pathways and modalities. Major cell death subroutines are as follows: **a |** Cell surface receptors (e.g., TNF family, Fas [280,281] induce extrinsic apoptosis via TRADD and caspases 3/7; they can also activate NF-κB for survival gene expression [282]. **b |** Intrinsic apoptosis is initiated by MOMP, which releases cytochrome *c* via BAX/BAK [283]. activating caspase 9 and downstream caspases 3/7 [284,285]. DAMPs include PGE2 and ATP. **c |** With caspase 8/IAP depletion/inhibition, cell surface receptors activate caspase 8, leading to MLKL-mediated necroptosis via RIPK1 [286,287]; NF-κB activation via MEKKs induces survival [288]. Proinflammatory DAMPs: HMGB1, ATP, histones, HSPs, exRNA, cfDNA, IL1α, IL33, IL6. **d |** ICD is initiated by ER stress. Receptors (e.g., TLRs, CD91) activate NF-κB and ISGs via TLR/STING signaling [92,289]; P2RX7 mediates ENTPD1/NT5E (abrogating immunogenicity) and activates caspases to release LAMP1/ROCK1 [290], driving immune stimulation and cell death (also via LAMP1/ROCK1-caspase cascades [13]). ICD DAMPs (CALR, HSPs, HMGB1, ATP, ANXA1, dsRNA, dsDNA, IFN-I) support antitumor immunity. **e |** MPT-driven necrosis is triggered by severe oxidative stress and Ca^2+^ overload [291]. Drugs (e.g., acetaminophen, metformin) induce Gadd45β; downstream JNK interacts with mitochondrial Sab to release ROS [292], cooperating with p53 in MPT-driven apoptosis. DAMPs: HAX1, ATP. **f |** Ferroptosis arises from iron overload and lipid peroxidation [293], induced by TFR or glutathione deprivation. Mediated by GPX4 inactivation or increased Fe^2+^ [294,295], promoting ROS release and cell death [296]. DAMPs identified in ferroptosis all have proinflammatory functions. Figure created with Bio GDP.

The Nomenclature Committee on Cell Death (NCCD) classifies cell death into accidental (uncontrolled, physicochemically triggered) and regulated cell death (RCD, genetically programmed for homeostasis) [120,121]. Only RCD modalities with ordered release of DAMPs—such as calreticulin (CRT) exposure prior to membrane rupture—induce ICD, a process critical for cancer clearance (Fig. 3) [[121], [122], [123]].

ICD, a functional immune outcome marked by antigen-specific T cell activation against dying cell antigens, is triggered by therapies including OV infection, anthracyclines, RT, and targeted agents [120,124]. These inducers share endoplasmic reticulum (ER) stress as a core mechanism, driving sequential DAMP dynamics: early CRT exposure recruits immune cells, while late release of HMGB1, ATP, and heat shock proteins (HSPs) amplifies responses [125]. As known ICD inducers, OVs uniquely modulate the TME by triggering DAMP release from dying tumor cells, activating innate/adaptive immunity—with efficacy varying by viral type (e.g., Ads vs. HSV) due to distinct replication kinetics [120,[126], [127], [128]].

ICD immunogenicity depends on antigenicity (tumor antigen recognition) and adjuvanticity (immune activation) [129]. Under homeostasis, cellular turnover is non-immunogenic, but OV-induced DAMPs act as "danger signals" to trigger pro-inflammatory/cytotoxic responses [14,130]. These DAMPs interact with PRRs (e.g., TLRs, cGAS-STING) to defend against infection and boost antitumor immunity, initiating tumor antigen-specific responses [64,131]. This section focuses on how OV-induced ER stress and reactive oxygen species (ROS) regulate tumor cell immunogenicity, key DAMP (CRT, ATP, HMGB1) mechanisms, and how OV-specific pathways (e.g., PERK/eIF2α-mediated ER stress) complement conventional ICD induction [81,[132], [133], [134]].

### 4.1 ER stress and ROS generation

**Fig. 4:**
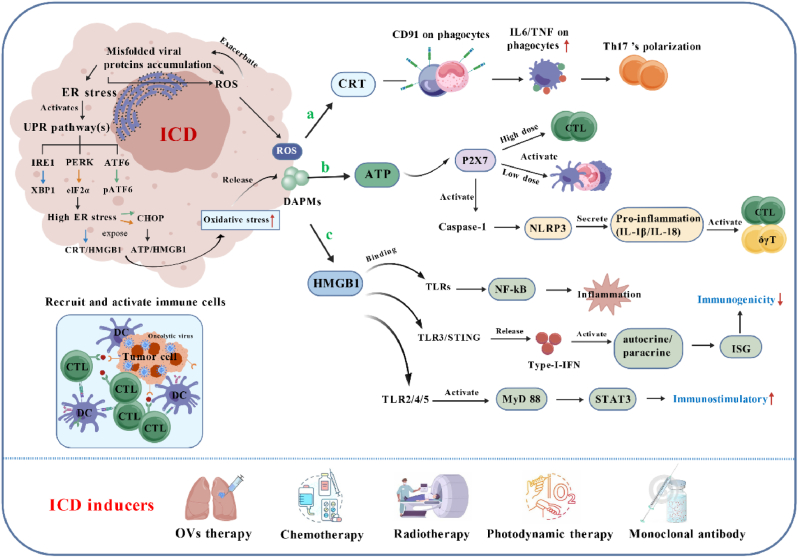
Mechanism of ICD induced by dying tumor cells. ICD is key for enhancing antitumor effects; inducers include chemotherapy [297], RT [125], and OVs [14,235,298]. Viral infection impairs ER protein-folding, causing misfolded protein accumulation [299] triggering ER stress and activating three UPR pathways: IRE1 mediates XBP1 splicing (ER homeostasis); PERK phosphorylates eIF2α (translation attenuation); ATF6 translocates to the nucleus (ER chaperone induction). Sustained ER stress overwhelms these responses, exposing CRT, HMGB1, and ATP. Subsequent ROS/oxidative stress pathways drive release of ROS and DAMPs, shifting tumor cells from survival to death programs [300]. **a |** CRT (a major DAMP on the surface of dying cells relies on PERK-mediated eIF2α phosphorylation [145]. It interacts with CD91 on phagocytes, promoting IL-6 and TNF expression in DCs and triggering pro-inflammatory Th17 polarization [146]. **b |** ATP is released extracellularly via vesicles, binding to the ionotropic receptor P2X7 (in a concentration-dependent manner) to activate cytotoxic T cells, promote DC activation, and recruit macrophages and neutrophils [153,159]. It also induces caspase-1-dependent activation of the NLR family, driving inflammasome-mediated secretion of IL-1β and IL-18 (pro-inflammatory) and ultimately activating CTLs and γδ T cells [160,162]. **c |** HMGB1 is released by activated macrophages or dying tumor cells, binding to TLRs to activate NF-κB and induce inflammation [164,169]. The TLR3/STING pathway releases IFN-I to activate autocrine/paracrine signaling, thereby inhibiting immunogenicity [301]; additionally, TLR4 triggers the MyD88 pathway during ICD to exert immunostimulatory effects [302]. Figure created with Bio GDP.

ER stress and ROS are conserved ICD drivers. OVs hijack the ER (for viral protein folding) in metabolically active tumor cells, causing misfolded protein accumulation and ER stress [49,135]. OV replication also disrupts mitochondria (e.g., NDV, MeV rely on mitochondrial ATP and NADPH), while ER stress activates IP3R channels to impair mitochondrial electron transport chains, generating ROS [136,137]. ROS oxidizes ER-resident protein disulfide isomerase (PDI), forming an "ER stress-ROS" vicious cycle that promotes DAMP release (e.g., CRT exposure, HMGB1/ATP secretion) (Fig. 4) [49,[138], [139], [140]]. Eukaryotic cells counter ER stress via the unfolded protein response (UPR, initiated by IRE1α, PERK, ATF6) [141,142]. Severe stress activates IRE1α-CHOP to exacerbate cell death [142]; PERK phosphorylates eIF2α to reduce ER protein load and induce CHOP [143], and under extreme stress, PERK-mediated eIF2α phosphorylation further promotes the surface exposure of CRT and HSPs [144].

### 4.2 CRT

CRT is an ER chaperone regulating calcium homeostasis and signal transduction; its core ICD role is acting as an "eat-me" signal, regulated by OV-induced ER stress [145]. OV infection triggers sustained ER stress (e.g., viral protein overload), driving CRT translocation from the ER to the plasma membrane via the PERK-eIF2α-ATF4-CHOP axis [20,146]. Surface-exposed CRT binds DC-expressed CD91 (LRP1) [147], enhancing DC phagocytosis of dying tumor cells and cross-presentation of TAAs to CD8^+^ T cells, activating tumor-specific CTLs (Fig. 4a) [49]. Multiple OVs (HSV-1 [148], Ads [149], VACV [150]) induce CRT exposure via this pathway. CRT also synergizes with HSPs: CD91 binds CRT/HSP90, and DC-expressed CD40 interacts with HSP70, further boosting CTL activation [151,152].

### 4.3 ATP

Extracellular ATP is a key "find-me" signal in ICD that exerts its role via secretion—this process depends on the apoptotic stage of tumor cells and OV-induced stress [153]. During OV-induced ICD, ATP is released via (1) viral replication-enhanced membrane permeability (e.g., pore formation) and (2) ROS-activated pannexin-1 channels [49,154]. OVs like RV [155,156], MeV [136], and NDV [137] drive TME ATP accumulation, correlating with replication efficiency. Extracellular ATP binds immune cell purinergic receptors (notably P2X7) [[157], [158], [159]], promoting macrophage/DC maturation, cytokine (IL-1β, TNF-α) production [160,161], NLRP3 inflammasome activation (IL-1β-mediated immune recruitment) [162,163], and CTL/neutrophil infiltration (Fig. 4b) [156]. TME ectonucleotidases (CD39, CD73) hydrolyze ATP to immunosuppressive adenosine, balancing immune activation.

### 4.4 HMGB1

HMGB1 is a nuclear protein that maintains chromatin structure; its role in ICD is to amplify inflammation, and this function is activated by OV-mediated tumor lysis [[164], [165], [166]]. OV infection (e.g., Ads, NDV [167], Coxsackievirus [CV] [168,169]) causes membrane damage, driving HMGB1 passive release from the nucleus to the TME [166]. Released late in cell death, HMGB1 sustains immune responses via (1) activating DCs to prime tumor-specific T cells; (2) suppressing Tregs to enhance CD8^+^ T cell cytotoxicity [170,171]; (3) attracting DCs/Th1 cells and regulating DC migration to lymph nodes [172]; (4) binding DC TLR4 to activate NF-κB/IFN pathways, driving pro-inflammatory cytokines (IL-6, IFN-γ) and a positive feedback loop (Fig. 4c) [49,173,174]. Clinically, this manifests as OV-infected tumor erythema and swelling, correlating with immune infiltration.

In summary, CRT exposure (antigen presentation initiation), ATP release (immune recruitment), and HMGB1 secretion (inflammation sustainment) form an "ICD signaling axis" in OV-induced ICD [13]. Triggered by OV-mediated lysis and DAMP release, this axis activates innate/adaptive immunity [20,175,176]. These DAMPs serve as OV efficacy biomarkers and OV engineering targets (e.g., enhancing CRT translocation) [14]. Maximizing ICD via OV engineering to overcome immunosuppressive TME is key to boosting antitumor potency [7].

## 5 Engineering OVs enhances antitumor efficacy

Naturally occurring OVs, such as MeV, NDV, and RV, exhibit inherent tumor-targeting properties [[177], [178], [179]]. However, their clinical efficacy as monotherapies remains limited, primarily due to suboptimal oncolytic potency and restricted immunostimulatory activity. Consequently, genetic engineering has become a pivotal strategy for enhancing the tumor selectivity and antitumor immunity of OVs [180]. Current engineering strategies aim to either augment safety and tumor selectivity (e.g., by deleting viral virulence genes like HSV-1 ICP34.5 to reduce neurotoxicity or deleting VACV TK to restrict replication) or to enhance antitumor immunity (e.g., by arming viruses with immunomodulatory transgenes such as GM-CSF) [[181], [182], [183]]. These strategic modifications, particularly in combination with other therapies, have improved therapeutic outcomes. The following section highlights selected OVs—either clinically approved or under investigation—whose unique biology has enabled diverse engineering approaches to boost oncolytic potency (Box 1).

Box1 **Characteristics of viruses Developed as oncolytic virus agents**

Newcastle disease virus (NDV)

NDV is an RNA virus belonging to the Paramyxoviridae family, with a spherical morphology and a genome length of approximately 15.2 kb [316]. According to the severity of NDV-induced diseases, it can be classified into three categories: lentogenic (nontoxic), mesogenic (intermediate), and velogenic (highly toxic). Virulence is mainly determined by sequence variation in the F gene; highly fusogenic F proteins expressed on the surface of infected cells efficiently form syncytia to facilitate viral propagation from cell to cell [274,317].

Adenovirus (Ads)

Ads is an envelope-less DNA virus with icosahedral symmetry, consisting of up to 15 structural proteins [318]. Because of its large linear double-stranded genome (spanning 26–45 kb), longer DNA sequences can be loaded for diverse genetically engineered modifications [319]. Ads are classified into seven subtypes (A-G), of which group C adenoviruses are non-oncogenic; serotypes 2 and 5 in particular have been evaluated as potential oncolytic agents and extensively studied for gene therapy applications [320].

Herpes simplex virus (HSV)

HSV is an enveloped double-stranded DNA virus belonging to the Herpesviridae family, with a large dsDNA genome (152 kb) encapsidated in an enveloped icosahedral capsid [321]. It predominantly infects neuronal cells, replicates in the nucleus, and does not cause insertional mutagenesis when genetically modified—attributes that have made HSV-1 a favored candidate for oncolytic virus development [322]. Based on their replication strategies, genetically engineered HSV-1 vectors are categorized into three groups: (i) replication-defective, (ii) conditionally replicating, and (iii) amplicon vectors. Among these, conditionally replicating HSV-1 vectors are designed to specifically target cancer cells. In addition, they have also been engineered to express therapeutic transgenes to enhance anticancer immunity [323].

Coxsackievirus (CV)

CV is a non-enveloped, single-stranded RNA virus belonging to the Enterovirus genus of the Picornaviridae family. It possesses an icosahedral capsid approximately 30 nm in diameter [324]. The viral genome consists of a positive-sense single-stranded RNA (+ssRNA) strand of ∼7.5 kb, which plays essential roles in viral replication and protein synthesis [325]. However, due to its relatively compact genome size, CV has limited capacity for accommodating exogenous genetic sequences. Therefore, careful optimization of transgene size and insertion sites is required to enhance therapeutic efficacy while maintaining viral viability [326].

Vaccinia virus (VACV)

VACV is an enveloped double-stranded DNA (dsDNA) virus, a member of the Poxviridae family (Orthopoxvirus genus), that replicates exclusively in the cytoplasm and is a naturally occurring oncolytic virus [327]. VACV has a large genome (∼190 kb) and can insert and stably express exogenous therapeutic genes of at least 25 kb in a single vector [328]. In addition, it exhibits broad tumor tropism and can be administered systemically, entering target cells via a membrane fusion pathway without requiring specific cell surface receptors [35,329].

Measles virus (MeV)

MeV is a negative-sense single-stranded RNA (ssRNA) virus belonging to the Morbillivirus genus of the Paramyxoviridae family. Its ∼16 kb genome encodes six structural proteins and two non-structural proteins [330]. It exhibits natural lymphotropism in humans and primates, where it replicates in lymphoid tissues and induces immunosuppression. Notably, attenuated yet replication-competent MeV vaccine strains demonstrate inherent oncotropism (tumor tropism) and have emerged as promising candidates for oncolytic virotherapy [36].

Reovirus (RV)

RV is a naturally occurring non-enveloped virus, one of nine genera belonging to the Reoviridae family. Its genome contains 10 segments of double-stranded RNA (dsRNA), replicates preferentially in cancer cells, and exhibits oncolytic activity both in vitro and in vivo [331]. In addition, it has an innate interferon-inducing ability to harness the host immune response to enhance antitumor activity in vivo, and has been shown to have oncolytic potential against a wide range of cancer cell lines and isolated tumor tissues [332,333].

Alt-text: Box1

Initially, the primary objectives of recombinant OV engineering were to enhance target specificity, selective replication capability, and viral safety [1]. Key strategies involved: (1) Modifying viral surface proteins to enable specific binding to receptors overexpressed on tumor cells (e.g., EGFR, CD46, ICAM-1) or exploiting deficient defense mechanisms (e.g., impaired interferon signaling pathways) [184,185], as exemplified by Ads DNX-2401 which was engineered with modified fiber proteins to target integrins (αvβ3/αvβ5) for enhanced tumor specificity [186,187]; (2) Deleting viral genes essential for replication in normal cells to impose dependence on tumor-specific signaling pathways (e.g., p53 deficiency, RAS activation, or interferon signaling defects) [188], illustrated by VACV JX-594 where deletion of the thymidine kinase (TK) gene critical for DNA metabolism restricted replication to tumor cells [189]; and (3) Improving viral safety through deletion of virulence genes (e.g., ICP34.5 in HSV) or implementation of tissue-specific promoters [181]. These genetic modification strategies significantly improved the antitumor efficacy of OVs and advanced the field of genetic engineering. More recently, strategies focusing on inserting genes encoding pro-inflammatory cytokines, immune modulators, or tumor antigens into viral genomes to reverse the immunosuppressive TME and enhance immune activation have gained prominence [7,190]. Notable examples include HSV-based T-VEC and VACV-based JX-594, both engineered to express GM-CSF to mediate immune cell recruitment into the TME [181,191].

Advances in genetic engineering have cemented engineered OVs as a pivotal platform for developing next-generation cancer immunotherapies [38,[192], [193], [194]]. To date, four OV products have been approved worldwide for cancer treatment. These include the recombinant human Ads type 5 (H101), approved in China (2005) for advanced nasopharyngeal carcinoma patients unresponsive to conventional radiotherapy or chemoradiotherapy [195,196]; T-VEC, approved by the FDA (2015) for the local treatment of recurrent advanced melanoma [4,197]; Delytact (a modified HSV), approved in Japan (2021) for the treatment of malignant glioma [198]; and coxsackievirus A21 (CVA21), approved in Australia (2023) for melanoma treatment [199]. In addition to these clinical successes, a wide range of other viruses are under investigation as recombinant OV-based platforms, including NDV [61,113,183,200,201], Ads [82,195,[202], [203], [204], [205], [206], [207]], HSV [83,181,[208], [209], [210], [211], [212]], MeV [213,214], RV [215], VACV [182,216], and CV [217]. We summarize several representative engineered OVs, highlighting how their diverse modification strategies have enhanced antitumor efficacy. Despite existing limitations, these candidates provide a crucial theoretical foundation for advancing future OVs into clinical trials (Table 1).

## 6 ICD inducers enhance tumor immunity

ICD inducers represent a promising class of anticancer agents that not only exert direct tumor-killing effects but also activate the immune system to elicit antitumor responses [218]. Since the unique immunostimulatory properties of ICD were first characterized, its clinical significance in cancer therapy has been increasingly acknowledged. A wide range of stimuli capable of triggering ICD have been identified, including biological (OVs), chemical (chemotherapeutic agents like anthracyclines, paclitaxel), and physical (RT, PDT) modalities [19,64]. These inducers share a common ability to activate ER stress pathways—ultimately driving DAMP release to initiate ICD—while exhibiting distinct mechanistic profiles: some induce ER stress as a secondary effect of perturbing tumor cell homeostasis, whereas others trigger ER stress through cytoplasmic ROS generation or selective targeting of the endoplasmic reticulum to disrupt its homeostasis [13]. We summarize the major therapeutic modalities known to induce ICD, most of which have entered clinical use or are under clinical evaluation (Box 2). While single-agent ICD inducers may have limited efficacy in achieving in situ tumor ablation and establishing long-term antitumor immunity, exploring the unique attributes of diverse ICD inducers and their roles in antitumor responses remains crucial for optimizing tumor immunity and advancing combination therapeutic strategies.

Box2 **The classes and characters of ICD inducers.**

Oncolytic viruses

ICD is the main mechanism by which OVs exert their antitumor effects. Compared to other ICD inducers, OVs have the advantage of selectively killing tumor cells without harming healthy cells, remodeling the TME, and establishing effective antitumor immune responses [334]. Natural OVs can kill tumor cells, but their safety and efficacy need improvement; whereas genetically engineered viruses reduce viral toxicity and improve safety, with significantly enhanced tumor-targeting and oncolytic ability [335]. For example, newly developed recombinant NDV strains can effectively promote tumor cell death by inducing systemic antitumor immunity, and have demonstrated significant antitumor effects in clinical trials [61].

Chemotherapy drugs

Chemotherapy uses drugs that inhibit or kill tumor cells at different stages of their growth and proliferation. Various clinically used chemotherapy drugs—such as anthracyclines, platinum-based drugs, alkylating agents, and paclitaxel—have been identified as ICD inducers [297]. Chemotherapy-induced ICD exerts effects by synergizing tumor cell killing (via chemotherapy activity) and activating antitumor immune responses, thereby achieving better therapeutic outcomes [336]. A key feature of this process is the surface exposure of CRT and release of ATP/HMGB1 (signature molecules of ICD) accompanying chemotherapeutic drug-induced apoptosis—this is common to all ICD-inducing chemotherapy drugs [337].

Radiotherapy (RT)

RT uses ionizing radiation to damage dsDNA and directly induce cell death. Meanwhile, the abscopal effect (distant effect) of radiotherapy induces ICD by upregulating MHC class I molecules and apoptosis-associated molecules to drive immune responses [297]. The efficacy of RT-induced ICD depends on radiation type, radiation dose, and tumor type. In addition, RCD (regulated cell death) modalities such as necroptosis and ferroptosis can enhance the radiosensitivity of tumor cells [225,338]. Therefore, RT is often used as adjuvant therapy combined with other ICD inducers to produce synergistic antitumor effects.

Photodynamic Therapy (PDT)

PDT is a non-invasive tumor treatment that activates photosensitizers (PS) via laser irradiation to generate ROS by altering the oxygen state, enabling precise targeting of tissues [298]. Compared with traditional treatments, PDT is less invasive and less likely to damage normal tissues. The immune response induced by PDT is mediated by inducing ICD, destroying immune-restricted tumor tissue, and triggering a chain reaction that “warms up” the TME [339,340].

Monoclonal antibodies (MAb)

MAbs are immunoglobulins designed to target specific epitopes on antigens, with main mechanisms including ADCC, CDC, and antibody-dependent cellular phagocytosis (ADCP) [235,341]. For example, cetuximab induces ICD by directly triggering ER stress on the cell surface to promote DC phagocytosis; it also indirectly induces the polarization of tumor-associated macrophages (TAMs) by inhibiting IL-6 expression via the NF-κB and STAT3 pathways [342]. In addition to inducing ICD as monotherapy, MAbs can be combined with other immunotherapies or chemotherapies to induce more potent ICD and enhance the tumor-killing effect.

Alt-text: Box2

## 7 Enhanced immunotherapy via OVs combined with ICD inducers

**Fig. 5:**
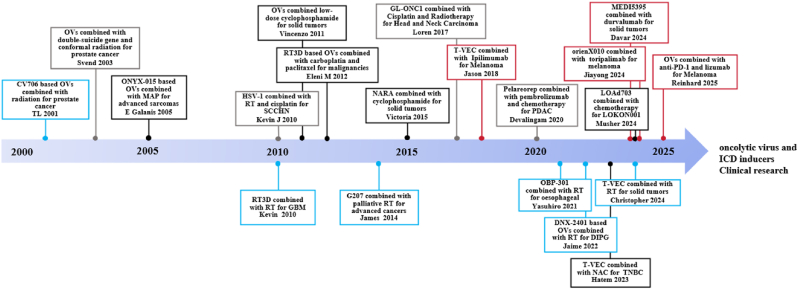
Timeline of clinical research on OVs and ICD inducer combined immunotherapy. Timeline of clinical research on OVs combined with ICD inducers: The concept of ICD was first proposed in the 2000s, and two OVs were approved for cancer treatment during this period. Since then, increasing studies have identified that other therapies (including OVs themselves) can induce ICD, prompting researchers to combine OVs with ICD inducers for cancer treatment. This figure summarizes key research on combining OVs with different types of ICD inducers, ranging from exploratory studies in the 2000s to current findings confirming that combined treatment significantly improves cancer patient survival. These combinations include OVs plus chemotherapy (black) [221,222,[303], [304], [305], [306]], OVs plus RT (blue) [231,[307], [308], [309], [310], [311]], OVs plus targeted formulations (red) [79,241,261,312], and triplet combinations (gray) [245,[313], [314], [315]].

As key inducers of ICD, OVs eliminate tumors via viral replication-triggered ICD (DAMP release, e.g., CRT, ATP) and TME remodeling—distinct from conventional therapies’ non-specific cytotoxicity [14,16]. OV monotherapy faces bottlenecks: insufficient ICD (poor spread in TME with Tregs/MDSCs) and weak systemic immunity (failure to induce long-lived memory T cells to target metastases). These make OVs ideal for combination with ICD-inducing modalities (chemotherapy, RT, targeted agents), which not only compensate for OV weaknesses but also create synergistic "chain reactions" (rarely emphasized in literature). Timeline of clinical research on immunotherapy confirms the feasibility of combining OVs and ICD inducers, though clinical translation requires addressing safety profiles and unresolved biological questions (Fig. 5).

### 7.1 OVs in combination with chemotherapy

Chemotherapy kills rapidly dividing cells via class-specific DNA damage (anthracyclines: intercalation; alkylating agents: cross-linking; antimetabolites: nucleotide disruption), and its synergy with OVs goes beyond TME remodeling [219]. Single-cell RNA sequencing shows chemotherapy transiently upregulates viral receptors (e.g., CD46 for MeV, CAR for Ads) 48–72 h post-treatment—creating an "infection window" for sequential OV use [220]. This explains why TNBC patients receiving T-VEC after paclitaxel/anthracyclines achieved 45.9 % RCB 0 (complete response) and 65 % RCB 0–1, 2–3-fold better than OV monotherapy (<20 % RCB 0–1) [221]. OVs also address chemotherapy's flaws: they convert "silent" cell death to ICD via DAMP release (fixing poor immunogenicity) and downregulate efflux pumps (e.g., ABC transporters) to reverse drug resistance—for example, LOAd703 inhibits treg-expressed A2AR, reducing immunosuppressive adenosine and boosting T-cell responses [222]. Yet, temozolomide (TMZ) combined with oHSV shows enhanced cytotoxicity in vitro but fails to improve survival in orthotopic glioblastoma models, even abrogating oHSV's beneficial effects when administered concurrently [223]. This antagonism may relate to TMZ-induced impairment of intratumoral T cells and macrophages—thus, sequential administration (TMZ first, followed by oHSV after 72 h to avoid T-cell suppression) has been proposed to reverse this effect. Personalized optimization requires prioritizing treatment timing (a key consideration highlighted in trials) to avoid antagonism, while synergistic myelosuppression (a known toxicity of TMZ, potentially exacerbated by OV-induced mild hematopoietic impairment) further demands integration of hematopoietic biomarkers into dosing regimens [224].

### 7.2 OVs in combination with radiotherapy

RT induces tumor death via double-strand DNA breaks, but its unique synergy with OVs lies in the epigenetic/metabolic co-regulation of ICD—a mechanism overlooked in previous trials. Specifically, RT upregulates viral receptors (e.g., coxsackie-Ads receptor [CAR]) and disrupts IFN signaling, leading to 2–3-fold higher OV replication [225,226]. Meanwhile, RT rewires TME nutrient metabolism: reduced glucose availability limits MDSC accumulation, and increased extracellular ATP enhances DC recruitment [[227], [228], [229]]. OVs activate antitumor immune responses through multiple mechanisms, among which interference with the CD39/CD73-adenosine pathway has been established as a critical immunostimulatory route [230]. Consistent with this, a Phase I study of esophageal cancer patients unfit for standard chemoradiation showed that this combination (OBP-301 plus RT) achieved a 91.7 % local objective response rate (8/13 CR, 3/13 PR), with histopathological evidence of increased CD8^+^ T-cell infiltration and PD-L1 upregulation [231]—findings that align with the immunostimulatory effects of CD39/CD73 pathway inhibition [232]. In addition, abscopal effects were not explicitly reported in this trial, though preclinical evidence suggests OV-mediated TME modulation could support distant antitumor responses [233]. The abscopal effect remains a "black box," however: while OVs are known to induce metastatic antigen cross-priming via DC migration to lymph nodes, clinical trials lack validated predictors of this effect [234]. Baseline T-cell clonality or IFN-γ levels show potential but remain unstandardized, making abscopal responses anecdotal and highlighting the need for "abscopal-predictive" diagnostic tools [226,233]. Safety risks reveal a paradox: OV-induced inflammation is required to support ICD but damages normal tissues. Future protocols should integrate inflammation modulators (e.g., low-dose IL-10) to balance ICD induction and tissue protection—better than limiting radiation volume, which compromises local control.

### 7.3 OVs in combination with targeted agents

Targeted agents offer precision modulation of oncogenic/immune pathways, with synergy defined by context-dependent immunometabolic crosstalk (unachievable with chemotherapy/RT) [235]. Tyrosine kinase inhibitors (TKIs) like sorafenib inhibit RAF/MEK/ERK signaling and VEGF receptors to boost OV replication by 5–10-fold and reduce TME hypoxia (VEGF inhibition) for better OV spread [236]. ICIs like pembrolizumab reverse T-cell exhaustion [237], but OVs add ICD-dependent checkpoint reversal—only ICD-positive tumors upregulate PD-L1, reducing off-target irAEs [238]. This explains the efficacy of CG0070 plus pembrolizumab in bladder cancer: the Phase 2 CORE-001 trial showed its 12-month CR rate was 57.1 % (intention-to-treat: 20/35, 95 % CI 40.7 %–73.5 %) vs. 15 %–20 % with ICI monotherapy. With a median follow-up of 26.5 months, the 24-month CR rate remained durable at 51.4 % (18/35, 95 % CI 34.9 %–68.0 %) [238]. A major unmet need is combination resistance: some patients develop OV-neutralizing antibodies despite ICIs, which is attributed to insufficient DC activation [239]. Preclinical work shows that OVs combined with TLR agonists (e.g., poly(I:C)) enhance DC maturation and reduce such OV-neutralizing antibodies by 30–40 %, a strategy tested in Phase I melanoma trials [240,241]. For TKI-resistant tumors, OVs induce synthetic lethality via downregulation of bypass pathways; this effect is lost in TP53-mutant tumors, highlighting TP53 as a companion biomarker [242]. Additionally, toxicity management requires nuance: TKI-OV-associated hypertension is driven by OV-induced endothelial activation, a risk that can be predicted by soluble VCAM-1 levels [243]; ICI-OV-associated dermatitis is linked to skin-homing T cells, requiring treatment with topical calcineurin inhibitors (not systemic corticosteroids, which blunt immunity) [244].

Combination strategies address OV limitations through distinct synergistic mechanisms: chemotherapy remodels TME for enhanced viral spread, RT boosts replication via DNA damage responses, and targeted agents precisely modulate immune pathways, although each of these modalities may have safety issues that require attention (Table 2). Future efforts should focus on personalized regimen design, incorporating biomarker-guided patient selection and rigorous safety monitoring to balance efficacy with tolerability. Ongoing clinical trials across various solid tumors continue to optimize these promising combinatorial approaches [[245], [246], [247]].

**Table 2:** Efficacy comparison of combined OVs and ICD inducers.

Combination Strategy	Study Population	Trial Phase	Key Efficacy Outcomes	Safety
ONYX-015 + Mitomycin-C, Doxorubicin, Cisplatin	Advanced Sarcomas	Phase I/II	ORR: 16.7 % (1 out of 6 patients achieved partial response [PR]) mPFS:NR	NR [303]
Ads + Cyclophosphamide	Advanced Solid Tumors	Phase I/II	DCR: 77 % (Oral + i.v. CP) vs 22 % (Virus only) mPFS: 376 d (Oral + i.v.) vs 63 d (Virus only)	Excellent. Only 2 Gr 3 events in 1 patient; no Gr 4–5 AEs [304]
			1-yr OS: 42 % (Oral + i.v.)	
RV (RT3D) + Carboplatin/Paclitaxel	Advanced Malignancies	Phase I/II	ORR: 26.9 % (7/26) mPFS: NR	Well-tolerated. Most toxicities were Grade 1–2 [305]
RV (RT3D) + Cyclophosphamide	Advanced Solid Tumors	Phase I	ORR/mPFS:NR	Well-tolerated overall; Gr 3/4 AEs only observed at/above MTD: 800 mg/m^2^ [306]
HSV(T-VEC) + Nonmetastatic TNBC	Triple-negative Breast Cancer	Phase II	ORR/mPFS:NR pCR (RCB0): 45.9 % 2-yr DFS: 89 %	T-VEC-related AEs: Common Gr 1–2 events, 4 thromboembolic events [221]
OVs LOAd703 + Chemotherapy	Unresectable/Metastatic Pancreatic Ductal Adenocarcinoma	Phase I/II	ORR: 44 % (8/18 patients with objective response) mPFS:NR	Most AEs: Gr 1–2.
				1 transient grade 3 aminotransferase elevation [222]
Ads(CV706) + Prior RT	Recurrent Prostate Cancer	Phase I	ORR/mPFS:NR	No irreversible Gr 3 or any Gr 4 toxicity, Gr 1 liver enzyme changes only [307]
RV(RT3D) + RT	Advanced Solid Tumors	Phase I	ORR: 28.6 % (20 Gy) to 71.4 % (36 Gy) mPFS/mOS: NR	Well-tolerated. No DLTs. Only Gr 1–2 toxicities [308]
Oncolytic HSV-1 G207 + RT	Recurrent Malignant Glioma	Phase I	ORR/mPFS:NR	Well-tolerated.
				No grade 3–5 AEs reported [309]
Ads (OBP-301) + RT	Esophageal Cancer	Phase I	ORR: 91.7 %	Well-tolerated. Transient, self-limited lymphopenia [231]
			Local CR: 8 patients	
			Clinical CR: 83.3 % (stage I) mPFS:NR	
Ads (DNX-2401) + RT	Paediatric Diffuse Intrinsic Pontine Glioma	Phase I	ORR: 25 % mOS: 17.8 months (vs < 12 mo expected)	Most AEs: grade 1–2; 1 case each of hemiparesis and tetraparesis [310]
			mPFS:NR	
HSV(T-VEC) + RT	Cutaneous Metastases	Phase II	ORR: 0 % mPFS: 2.5 mo (Combo) vs 1.2 mo (T-VEC)	Consistent with a well-known T-VEC profile. No new safety signals [311]
			mOS: 17.3 mo (Combo) vs 4.9 mo (T-VEC)	
HSV(T-VEC) + Ipilimumab	Advanced Melanoma	Phase II	ORR: 39 % (Combo) vs 18 % (Ipi alone)	Gr ≥ 3 AEs: 45 % (Combo) vs 35 % (Ipi). No new safety signals [79]
			OR: 2.9, P = .002 mPFS:NR	
orienX010 + Anti-PD-1 Toripalimab	Acral Melanoma	Phase II	Radiographic ORR: 36.7 %	Most AEs: grade 1–2; No Gr 3–5 AEs explicitly reported [241]
			-Pathological ORR: 77.8 % mPFS:NR	
NDV (IV MEDI5395) + Durvalumab	Advanced Solid Tumors	Phase I	ORR: 10.3 % (4/39 partial response [PR] mPFS:NR	Gr 3–4 AEs: 69.2 % (all), 30.8 % (virus-related) [261]
Gebasaxturev (CVA21) + Pembrolizumab	Resectable Melanoma	Phase I/II	pCR: 28 % (Combo) vs 40 % (Pembro alone)	Gr 3–4 TRAEs: 28 % (Combo) vs 7 % (Pembro). Manageable safety [312]
			ORR (RECIST): 32 % vs 27 %	
			mPFS:NR	
Ads + 3D-Conformal RT + Prodrug Therapy	Prostate Cancer	Phase I	ORR/mPFS:NR	Excellent. 94 % AEs were Gr 1–2 [313]
HSV-1 JS1/34.5^-^/47^-^/GM-CSF + Chemoradiotherapy	Squamous Cell Cancer	Phase I/II	ORR: 82.3 % (14/17 patients with tumor response) mPFS:NR	Well-tolerated. No Gr 3–5 TRAEs explicitly reported [314]
VACV (IV GL-ONC1) + Cisplatin + RT	Advanced Head and Neck Carcinoma	Phase I	ORR:NR mPFS:Not explicitly reported; 1-year PFS: 74.4 %, 2-year PFS: 64.1 % (median follow-up: 30 months)	Most AEs: Gr 1-2
				Gr 3 AEs: hypotension, mucositis, nausea/vomiting [315]
Pelareorep + Anti-PD-1 Pembrolizumab + Chemotherapy	Advanced Pancreatic Adenocarcinoma	Phase II	ORR:10 % (1/10 partial response [PR], duration: 17.4 months) mPFS:NR	Well-tolerated; most TRAEs Gr 1–2 (flu-like symptoms most common) [245]

## 8 Outlook

Substantial progress has been made in OV clinical development, with approved agents like T-VEC (melanoma) and Delytact (glioma) highlighting their potential to induce ICD and remodel the TME [4,197]. However, broader translation into diverse cancers and improved efficacy are hindered by unresolved challenges, context-dependent side effects, and technical limitations, which necessitate targeted innovations.

A primary challenge is inefficient systemic delivery. Intravenously administered OVs face antibody neutralization and clearance by the reticuloendothelial system (RES) [[248], [249], [250]]. Solid tumors present additional physical barriers: a dense extracellular matrix (ECM), as seen in pancreatic and desmoplastic breast cancers, impedes viral diffusion, while hypoxic regions downregulate key viral receptors, such as CD46 for MeV [251,252]. These delivery hurdles are linked to specific side effects. Preclinical studies show that intravenous administration of Ads-based OVs causes dose-dependent hepatotoxicity, evidenced by elevated ALT/AST in mice [253]. Furthermore, neurotropism remains a concern for CNS tumors even with engineered viruses like γ34.5-deleted HSV-1 [254]. Clinically, intratumoral injection of T-VEC induces grade 1–2 erythema and swelling in 60–70 % of melanoma patients [255], while systemic delivery of LOAd703 triggers chills in 33 % of patients [222].

To overcome these barriers, advanced delivery strategies are under active exploration. These include cell-based carriers (e.g., mesenchymal stromal cells, T cells) that shield OVs from immune recognition, and biomaterial-based approaches such as hypoxia-responsive nanovehicles (e.g., PEGylated liposomes) or mesenchymal stromal cells (MSCs) engineered to secrete matrix metalloproteinases (MMPs) to degrade the ECM [249,256,257]. Genetic engineering of OVs themselves, for instance using CRISPR to modify viral tropism by retargeting HSV-1 to glioma-specific EGFRvIII or Ads to ovarian cancer-overexpressed αvβ3 integrins, is another promising avenue to enhance tumor specificity and reduce off-target toxicity [258,259].

Beyond delivery, the host antiviral immune response presents a dual challenge. While IFN-I signaling is crucial for containing systemic viral dissemination and ensuring safety, it can also prematurely clear OVs within tumors, thereby curtailing replication and therapeutic efficacy [91]. Clinically, OV-induced IFN-I release manifests as grade 1–2 flu-like symptoms (e.g., fever, myalgia) in 30–50 % of patients receiving intravenous OVs [260]. These effects can be exacerbated when OVs are combined with ICIs, leading to increased fatigue and nausea [261]. Strategies to mitigate these effects include engineering OVs with tissue-specific miRNA target sites (e.g., liver-specific miR-122) or arming them with immunosuppressive transgenes like VEGF decoy receptors, which can dampen excessive innate immune activation without compromising antitumor immunity [7,262]. An alternative approach involves using tumor-specific promoters (e.g., the survivin promoter) to drive the expression of IFN-I antagonists locally within the TME, thereby preserving systemic antiviral defenses and antitumor T-cell function [211].

Tumor heterogeneity further complicates uniform therapeutic responses, introducing both spatial complexity—such as hypoxic cores that suppress viral replication and peritumoral regions rich in MDSCs that blunt immune activation—and temporal dynamics, including the selection of IFN-I-proficient tumor clones that drive acquired resistance [[263], [264], [265]]. Addressing this heterogeneity requires personalized strategies, such as using CRISPR to insert patient-specific neoantigens into OVs like NDV or employing patient-derived tumor organoids to pre-screen for the most effective OV variant [61,77,266]. Rational combination therapies are also vital; these extend beyond traditional ICIs to include agents like CD73 inhibitors that block the production of immunosuppressive adenosine in the TME [267]. Furthermore, optimizing treatment schedules, such as sequential administration of chemotherapy followed by OVs and then ICIs, has been shown to outperform concurrent regimens by avoiding antagonism [245].

Complementing the efforts to overcome biological barriers, the identification of reliable predictive biomarkers is critical for patient stratification. Current candidate biomarkers, including intratumoral CD8^+^ T-cell density, baseline interferon signatures, and viral receptor expression profiles, lack standardization and universal applicability. For instance, while CD8^+^ T-cell density predicts response to T-VEC in melanoma, it fails to do so in glioblastoma, likely due to the unique immunosuppressive constraints of the central nervous system TME [268]. Moreover, single biomarkers are insufficient to capture the complex crosstalk between the virus and the host immune system; a high IFN-I signature, for example, can correlate with either rapid viral clearance or enhanced antitumor immunity in different contexts [269]. Future directions should focus on integrating multi-omics data (e.g., single-cell RNA sequencing of the TME combined with dynamic monitoring of circulating viral DNA) to develop robust, OV-specific biomarker panels [77,270], alongside regulatory efforts to standardize detection methodologies.

Despite these advances, several translational limitations persist. The batch-to-batch production variability of engineered OVs increases the cost and complexity of personalized approaches, potentially delaying patient access [271]. There is also a need for longer-term safety monitoring, as late-onset side effects like autoimmunity may be underreported and require extended follow-up (e.g., ≥5 years for T-VEC) [272]. Additionally, the predictive power of current preclinical models is limited, as they often overestimate efficacy due to inherent differences from human tumors, such as lower infiltration of immunosuppressive cells [273]. Interdisciplinary innovations offer promising solutions to these challenges, including AI-driven models for predicting viral tropism and optimizing transgene design, and the development of stable, low-cost formulations like lyophilized NDV to improve accessibility in resource-limited settings [201].

In summary, while OV monotherapy provides clinical benefit for a subset of patients, its full potential will be realized through rational combinations and precision engineering. Successfully optimizing delivery platforms, advancing personalized OV constructs, validating predictive biomarkers, and resolving issues of scalability and long-term toxicity will be critical to expanding the impact of OV-based immunotherapy—moving decisively beyond "one-size-fits-all" approaches toward regimens meticulously tailored to the unique biology of each patient's tumor and immune system.

## CRediT authorship contribution statement

**GuoXiu Cao:** Writing – review & editing, Writing – original draft. **Chan Ding:** Writing – review & editing. **Jun Dai:** Writing – review & editing. **Xusheng Qiu:** Writing – review & editing.

## Ethics approval and consent to participate

Not applicable.

## Consent for publication

Not applicable.

## Availability of data and materials

Not applicable.

## Funding

This work was supported by the 10.13039/501100001809National Natural Science Foundation of China (32272978), Guizhou Provincial Natural Science Basic Research Program (No. QianKeHe Basic MS- [2025] 383), Guizhou Provincial Natural Science Basic Research Program (No. QianKeHe Basic- [2024] Youth 316), Zunyi Science and Technology Project (NO. [2024] 322) and Qian Ke Ping Tai Project (NO. [2021]1350-057).

**Table:** Abbreviations

OVs	Oncolytic viruses
TAA	Tumor-associated antigens
DAMPs	Damage-associated molecular patterns
PAMPs	Pathogen-associated molecular patterns
APCs	Antigen-presenting cells
ICD	Immunogenic cell death
ICIs	immune checkpoint inhibitors
TME	Immune microenvironment
NDV	Newcastle disease virus
RV	Reovirus
VACV	Vaccinia virus
CTL	Cytotoxic T lymphocyte
PDT	Photodynamic therapy
TME	Tumor microenvironment
ADCC	Antibody-dependent cell-mediated cytotoxicity
CDC	Complement-dependent cell-mediated cytotoxicity
NK	Natural killer cells
TLRs	Toll-like receptors
SFV4	Semliki Forest Virus 4
MYD88	Myeloid differentiation primary-response protein 88
TIRAP	TIR domain-containing adaptor protein
TRIF	TIR domain-containing adaptor protein inducing IFN-β
GM-CSF	Granulocyte-macrophage colony-stimulating factor
TRAM	TRIF-related adaptor molecule
SARM	Sterile α- and armadillo-motif-containing protein
NF-κB	Nuclear factor kappa-B
IFN- I	Type I interferons
EGFR	Epidermal growth factor receptor
RTK	Receptor tyrosine kinase
VEGF	Vascular endothelial growth factor
ERK 1/2	Extracellular signal-regulated kinases 1/2
BCL	B cell lymphoma
JAK1	Janus kinase 1
STAT	Signal transducer and activator of transcription proteins
IRF9	Interferon regulatory factor 9
ISGF3	Interferon-stimulated gene factor 3
NCCD	Nomenclature Committee on Cell Death
RCD	Regulated cell death
HMGB1	High mobility group box 1
CRT	Calreticulin
ATP	Adenosine triphosphate
HSPs	heat shock proteins
PRRs	pattern recognition receptors
ER	Endoplasmic reticulum
ROS	Reactive oxygen species
UPR	unfolded protein response
IRE1	inositol-requiring enzyme 1
TIR	Toll/IL-1 receptor
PERK	PKR-like ER kinase
ATF6	activating transcription factor 6
eIF2α	eukaryotic initiation factor 2α
CHOP	CCAAT/enhancer-binding protein homologous protein
TNF-α	Tumor necrosis factor-α
IL-6	Interleukin-6
STING	Stimulator of interferon genes
PDI	Protein disulfide isomerase
BBB	Blood-brain barrier
CNS	Central nervous system
MeV	Measles virus
CV	Coxsackievirus
DCR	Disease control rate
TK	Thymidine kinase
H101	Human adenovirus type 5
T-VEC	Talimogene laherparepvec
TRADD	TNFRSF1A associated via death domain
FADD	Fas associated via death domain
TRAF2/5	TNF Receptor Associated Factor 2/5
cIAPs	Cellular inhibitor of apoptosis;
cFLIP	CASP8 and FADD like apoptosis regulator
RIPK1	Receptor interacting serine/threonine kinase 1
BAX	BCL2 associated X
RIPK1	Receptor interacting serine/threonine kinase 1
MLKL	Mixed lineage kinase domain like pseudokinase;
p53	Tumor protein p53
PUMA	P53-Upregulated modulator of apoptosis
LAMP1	Lysosomal-associated membrane protein 1
AGER	Advanced glycosylation end product-specific receptor
P2RX7	Purinergic receptor P2X7
PANX1	Purinergic receptor P2X7
NT5E	5'nucleotidase
ENTPD1	Ectonucleoside triphosphate diphosphohydrolase 1
ISG	IFN-stimulated genes
GPX4	Glutathione peroxidase 4
ROCK1	Roiled-coil containing protein kinase 1
Sab	SH3 domain-binding protein that preferentially associates with Btk
JNK	c-Jun amino-terminal kinase
MKK4	MAPK kinase 4
MAP3K	Mitogen-activated protein 3 kinase
MPT	Mitochondrial permeability transition
PTPC	Transition pore complex
GSH	Synthesis of glutathione
GPX4	Synthesis of glutathione peroxidase 4
GSSG	Oxidized glutathione disulphide
CYPD	Cyclophilin D
Atg5	Autophagy-related gene 5
NOCA4	Nuclear receptor coactivator 4
TFR	Transferrin receptor protein
PGE2	Prostaglandin E2
ATP	Adenosine 5′-triphosphate
HAX1	HCLS1 associated protein X-1
exRNA	Extracellular RNA
cfDNA	Cell-free DNA
dsRNA	Double-stranded RNA
dsDNA	Double-stranded DNA
IL1a	Interleukine 1 alpha
IL33	Interleukin-33
IL6	Interleukin-6
IL1b	Interleukine 1 beta
ANXA1	Annexin A1
NT5E	Noiled-coil containing protein kinase 1
RES	Reticuloendothelial system
ECM	Extracellular matrix
MSCs	Mesenchymal stromal cells
MMPs	Matrix metalloproteinases

## Declaration of competing interest

The authors declare that they have no known competing financial interests or personal relationships that could have appeared to influence the work reported in this paper.

## Data Availability

No data was used for the research described in the article.
